# Assessment of hypertension and diabetes among lecturers in a Ghanaian university: Setting lifestyle practices as precursors

**DOI:** 10.1371/journal.pone.0338910

**Published:** 2025-12-29

**Authors:** Joseph Kwasi Brenyah, Peter Twum, Elliot Tannor Koranteng, Joana Kyei-Dompim, Portia Boakye Okyere, Florence Brenyah, Barbara Gyapong Korsah, Eric Adjei Boadu, Christian Agyare

**Affiliations:** 1 Department of Global and International Health, School of Public Health, Kwame Nkrumah University of Science and Technology, Kumasi, Ghana; 2 Department of Health Policy, Management and Economics, School of Public Health, Kwame Nkrumah University of Science and Technology, Kumasi, Ghana; 3 Department of Medicine, Kwame Nkrumah University of Science and Technology, Kumasi, Ghana; 4 School of Nursing, Kwame Nkrumah University of Science and Technology, Kumasi, Ghana; 5 School of Public Health, Kwame Nkrumah University of Science and Technology, Kumasi, Ghana; 6 Department of Hospitality and Tourism Education, Akenten Appiah-Menka University Skill Training and Entrepreneurial Development, Kumasi, Ghana; 7 Department of Occupational and Environmental Health and Safety, School of Public Health, Kwame Nkrumah University of Science and Technology, Kumasi, Ghana; 8 Department of Population and Reproductive Health, School of Public Health, Kwame Nkrumah University of Science and Technology, Kumasi, Ghana; 9 Faculty of Pharmacy and Pharmaceutical Sciences, College of Health Science, Kwame Nkrumah University of Science and Technology, Kumasi, Ghana; Mae Fah Luang University School of Anti Aging and Regenerative Medicine, THAILAND

## Abstract

**Introduction:**

Research works relating to the lifestyle of students, non-academic staff, and communities have had considerable attention. However, studies on the lifestyles of university lecturers in Ghana and their association with the occurrence of hypertension and diabetes have received very scanty attention.

**Objective:**

This study assesses the modifiable lifestyle practices among lecturers in a Ghanaian university, and how these are associated with the occurrence of hypertension and diabetes.

**Methods:**

It was a cross-sectional study design with a quantitative approach involving 205 lecturers in a Ghanaian university. Data were analyzed using descriptive statistics and regression.

**Results:**

The study found that 50.73% and 33.66% of lecturers were overweight (25.0 < 30 Kg/m^2^) and Obese (<30 Kg/m^2^) respectively. Also, 97.07% and 85.85% % of Lecturers had normal fasting blood sugar (<5.6 mmol/L) and normal random blood sugar (<11.1mmol/dL) respectively. Again, 62% of lecturers eat breakfast and only 5.4% take fruits and vegetables of 5 servings per day/week. Moreover, 34.6% of lecturers are habitual late meal takers. Again, 30% of lecturers do not undertake any form of exercise. A Lecturer’s age was found to be statistically significant (p = 0.044). It was found that, lecturer aged over 50 years accounted for about 91.25%. Also, 18.54%, 16.10%, and 12.68% of lecturers in Health Sciences, Engineering, Agriculture, and Natural Resources had high blood pressure (systolic >130mmgH, diastolic >90mmgH). Lecturers from the Colleges of Health Science and Engineering were 4 times [COR: 4.11; 95% CI (1.30–12.95)] more likely to develop high blood pressure when compared to those in other Colleges. The prevalence of hypertension and diabetes among the university lecturers was found to be 54.6% and 17.07% respectively.

**Conclusion:**

The study concludes that modifiable lifestyles are precursors for the occurrence of hypertension and diabetes among the lecturers studied in a Ghanaian university.

## Background of study

Health promotion and prevention embodied in the concept of salutogenesis have received global attention from healthcare policymakers as a measure impacting limited resources for healthcare [1–4]. Lack of health preventive measures and good lifestyles may predispose individuals to diseases such as diabetes, hypertension, kidney conditions, and strokes, among others. Several studies relating to the lifestyle of students, non-academic staff, and communities have received considerable attention [5–10]. However, studies on the lifestyles of university lecturers and the connection to the occurrence of hypertension and diabetes have received very scanty attention even though higher educational status is believed to be inversely related to the occurrence of hypertension and diabetes. In recent times, available studies have reported that 57.18% of lecturers have high BP (>120 mmHg, > 80 mmHg) [11]. Another study in an urban university in Uganda reported 26.2% of hypertension among faculty [12]. Also, the study of Omorogiuwa et al. [13] found that the prevalence of diabetes was 9.8% with an incidental finding (unknown) contributing 6.1% among the faculty of a university in Nigeria. This paper, therefore, seeks to assess the modifiable lifestyle practices of lecturers in a Ghanaian university and the onset of hypertension and diabetes.

The study is underpinned by the Social Contract Theory. This theory, developed by Locke and Rousseau (1588–1704) highlights the mutual relationship between individual factors, behavior, and the environment. In its application to lecturers, the theory seems to suggest that lecturers’ beliefs about their ability to manage their health (self-efficacy), their social environment (colleagues, family), and their behaviors (e.g., exercise habits, dietary choices) all interact to influence their risk of developing hypertension and diabetes. Working as a university lecturer often comes with sedentary lifestyles such as spending long hours sitting at desks or in classrooms. This can contribute to weight gain and increased risk of hypertension and diabetes. For instance, the busy schedules of university lecturers can make it challenging to find time for regular exercise, healthy meal preparation, or stress management activities. In essence, the social contract theory provides a framework to integrate these elements to explain how individual perceptions, beliefs, and behaviors, in interaction with the unique context of university life, influence the development and progression of hypertension and diabetes in this profession.

Lifestyle can be understood as individuals’ behavior regarding the living conditions that inform their health experiences [14,15]. Accordingly, lifestyle is related to the human development trajectory of social, economic, political, and cultural factors in one bloc and risk factors of physical activities, dietary behavior, tobacco use, and alcohol consumption in another bloc [16–18]. University lecturers are part of the biological classifications, have social roles, are active players in the labor market, have political orientations, have diverse cultural issues, and are not exceptional to individuals who are implicated in physical inactivity, unhealthy dietary practices, excessive alcohol and tobacco use. Knowing the lifestyle of university lecturers regarding these variables is therefore essential and would inform policy on their health status and the occurrence of hypertension and diabetes.

In this research, modifiable lifestyles were categorized as issues on dietary intake (breakfast intake), skipping of meals, content of food intakes, eating times), physical activities (exercise status, frequency of exercises, intensity of workouts, mode of exercising), smoking status (categories of substances, frequency of smoking, tobacco intensity), occupational factors (hours of work, reporting and closing times, daily workloads, usage of daily break-out periods, management of work schedule tasks), alcohol status (categories of alcohol intakes, frequency of alcohol intakes and intensity of alcohol intakes).

Many studies have reported outcomes on lifestyle variables concerning university lecturers. For instance, a study conducted among university faculty members by Mohaghegh et. al. [19] found that 72.5% of the participants had low physical activity levels and only 18.8% had medium physical activity levels. Also, a study conducted by Klein et al., [20] among physician faculty at the University of Alberta found that only 47.2% mentioned undertaking energetic physical activity more than 3 days a week, while 33.0% mentioned moderate physical activity more than 3 days a week. Again, a study conducted in Brazil among university faculty found that, frequency and intensity of the physical activities culminated in improved physical performance of the professors. The study further reported that a low number of professors performed regular physical activities, so 42% of professors were at high risk of cardiovascular diseases [21]. In a gender-categorized study among university staff conducted in universities of the West Indies, the study found low levels of physical activity in 51.9% of males and 62.2% of females [22]. Smoking as a modifiable lifestyle remains a significant global health threat [23,24]. Though tobacco use has many forms, the predominant use is direct smoking. Some research outcomes have mentioned that smoking is prevalent among people of low socio-economic status [23,25]. Their findings imply that smoking is low among the educated population. This assertion is corroborated by the study outcome of Klein, Guenther & Ross [20] who found smoking to be rare among the physician faculty.

Alcohol consumption has been mentioned as a lifestyle that predisposes many people to non-communicable diseases. For instance, in the UK, alcohol is the 3^rd^ lifestyle risk factor for morbidity and mortality aside from smoking and obesity [26]. A study conducted at a UK university reported that 39% of academic respondents were habitual alcohol consumers [27]. Similarly, a study by Klein et al., [20] reported that 86.2% of physician faculty members consumed alcohol.

University lecturers need a prime health status to accomplish their responsibilities, such as teaching, research, postgraduate training, university administration, and community services. In achieving this, meal intake is key. Meal intake is considered a building block in the maintenance of the functional activity of the body and hence is linked to the quality of life of an individual. However, some individuals including lecturers skip meals such as breakfast, lunch, or supper which has become an aspect of their lifestyle [28]. Skipping meals is adversely associated with several biomarkers and cardiometabolic risk factors, including weight gain, dyslipidemia, blood pressure, insulin resistance, and diabetes [29].

Again, the quality and quantity of food consumed are another aspect of a modifiable lifestyle important to human life. Many research outcomes have linked the increase in NCDs to unwholesome dietary behavior such as patronage of fatty processed meats, saturated fat, refined grains, salt, and sugars [30–34]. Aside from many dietary intakes lacking a balanced diet, available evidence also portrays low or lack of fruit and vegetable intake [33]. A study conducted among university professors in the Melilla campus of the University of Granada found two eating patterns. These were identified as a ‘western pattern’ (characterized by the consumption of dairy products, eggs, meat, sausages, refined oils, butter, sugar, processed baked goods, sugar-containing beverages, and alcoholic drinks) and a ‘mediterranean pattern’ (characterized by consumption of olive oil, fish, fruits, nuts, vegetables, pulses, cereals, and honey) as reported by Lopez-Olivares et al. [34].

From the above narrations, modifiable lifestyle assessment is essential as primary prevention towards a healthy life [35]. In this logic, the academic setting is seen as an unexplored but key environment for the promotion of a healthy lifestyle, which may impact the transfer of knowledge to students and other bodies.

An assessment of the prevalence of hypertension and diabetes among lecturers in a Ghanaian university using their lifestyle practices is significant because it can reveal opportunities to improve their well-being, job satisfaction, and overall effectiveness, which can positively impact the quality of education they provide. By understanding how lifestyle choices affect lecturers’ physical and mental health, universities can implement targeted interventions and support systems to promote a healthier and more productive academic environment.

University lecturers are an important professional group for the development of every country. However, not much is known about their lifestyle practices concerning the occurrence of hypertension and diabetes. This study, therefore, attempts to assess the occurrence of hypertension and diabetes among lecturers in a Ghanaian university using their lifestyles as precursors. It is believed that the outcome of this study may inform policy on health measures among university lecturers.

## Materials and methods

### Study setting

This study was conducted at the Kwame Nkrumah University of Science and Technology (KNUST), in Kumasi-Ghana. The study involved six (6) Colleges from February-August, 2022.

### Study design and approach

This was a cross-sectional study designed to assess the lifestyle of lectures at KNUST as a precursor for the occurrence of non-communicable diseases. The study employed only a quantitative approach in which data was taken using standardized structured questionnaires.

### Study population

The study population involved lecturers in the University. The lecturers’ population per college showed that the College of Health Sciences was the highest while Agric/Natural Resources was the lowest. The lecturer population in the other colleges was fairly distributed [36].

### Sampling technique

A simple probability technique was used to pick the name of a college and the day/date to visit. Two sets of papers were folded with the names of colleges (set 1) and the day/date of the visit (set 2). One undisclosed paper was selected from each set and the name of the college and the day/date to visit were harmonized. With this procedure, the College of Humanities & Social Sciences, College of Agric and Natural Resources, College of Art & Built Environment, College of Engineering, College of Science, and College of Health Sciences were the order of visit. We then used the ‘walk-in” system to select the study participants. Within the days of visiting a college, any lecturer we met in his/ her office was a prospective study participant.

### Sample size calculation

The formulae of Yamane [37] were used to calculate the sample size below:


$$n\;\;=\;\;\;\frac{N}{\text{1}+N(e^{\text{2}})}$$

where n = is the population of Lecturers at KNUST

E = is the level of precision

Therefore: $\text{n}=\frac{\text{838}}{\text{1}+\text{838}\;(\text{0}.\text{0025})}$


$$\text{n}=\frac{\text{838}}{\text{1}+\text{2}.\text{098}}$$

$$\frac{\text{838}}{\text{3}.\text{095}}$$

n=270

The study involved 205 participants. We could not use the 270 participants due to budget constrained especially relating to the biochemical test. Simple proportions were applied to secure the sample of lecturers in each college.

### Inclusion and exclusions criteria

Inclusion criteria were made up of the following:

1. All lecturers of KNUST who are in active service.
2. All lecturers in the departments selected under each of the colleges.
3. All Lecturer who consented to participate to participate in the study.

The exclusion criteria were made up of the following:

1. All lecturers who were employed in less than 6 months (probation period)
2. All lecturers who did not consent to participate in the study.

### Recruitment procedure

After using the simple random sampling to select the colleges, we then used the ‘walk-in” system to recruit the study participants. Lecturers were approached in their various offices. The purpose of the study was explained to them. Participants’ information leaflets were given. Lecturers who consent to participate were given consent forms to sign and date.

### Data collection tool

Data was collected using structured questionnaires. The questionnaires covered dietary habits, alcohol intake habits, physical activity status, and tobacco use. The questionnaire also captured anthropometric measurements (weight, height, blood pressure), biochemical parameters (blood glucose test), socio-demographic characteristics, and other lifestyle variables.

### Pre-testing

The data collection tool was pre-tested at the Kumasi Technical University which shares similar characteristics with Kwame Nkrumah University of Science and Technology and both universities are situated in the same city in Ghana.

### Data management

Only the researchers had sole access to the data. Data collected were cleaned and input into a Microsoft Excel spreadsheet. After checking for accuracy, the data is then imported into STATA version 14.0 (Stata Corp LP, College Station, Texas, USA) for statistical analysis.

### Study variables

Blood pressure (BP) and blood glucose (FBS/RBS) status were selected as the dependent variables. The BP calibrated as **Normal BP** was (≤ 120/80 mmHg); **Elevated PB (**Systolic between >120–129 mmHg and diastolic ≤ 90 mmHg); **Hypertension** (Systolic ≥ 130 or diastolic ≥ 90). The blood sugar was categorized into fasting blood sugar (FBS) and random blood sugar (RBS). Lecturers with FBS < 5.6 mmol/L, 5.7–6.7 mmol/L, and ≥ 7.0 mmol/L were classified as normal, pre-diabetic and diabetic. Lecturers with RBS of <11.1mmol/dL and >11.2mmol/dL were classified as normal and diabetic. Independent variables were sociodemographic; gender, age, marital status, staff rank, and lecturer’s colleges (categorized into binary variables; COHS/COS/COE and CABE/CANR/COHSS), family history of hypertension and diabetes and lifestyle.

### Data analysis

The study employed descriptive statistics by employing frequencies with their respective percentages. Also, the associational status was assessed using Chi-square (χ^2^) or Fisher’s exact tests where appropriate with a p ≤ 0.05 as a statistically significant level. Both bivariate and multivariate logistic regression analyses were executed and adjusted for colleges’ effects to find associations between blood pressure (BP) and independent variables. Predictors with significant association with BP in the logistic regression models were set at p ≤ 0.05 with a 95% confidence interval (95% CI) for both unadjusted and adjusted odds ratios (UOR, AOR).

### Ethical considerations

The study sought ethical clearance from the Committee on Human Research, Publication, and Ethics (CHRPE)-KNUST with approval reference no: CHRPE/AP/581/21. The objective of the research was explained to the study respondents. Consented participants were made to sign consent forms. Again, respondents were assured of confidentiality. Respondents were informed they have the right to withdraw from the study at any time they wish.

## Results

### Sociodemographic characteristics of lecturers

The study participants were 205 including 176 (85.9%) males. The mean age was 46.3 ± 9.4SD years with a range of 27–73 years. A majority (39.0%) were in age category 50 and above. The study found that 173 (84.4%) participants were married, and most of them were senior lecturers and lecturers representing 76(37.1%) and 75 (36.6%) respectively. The participants were fairly representative of all the colleges of the KNUST as shown in Table 1.

**Table 1:** Sociodemographic characteristics of respondents.

Factors	Freq. (n = 205)	Percent. (%)
**Gender**		
Male	176	85.85
Female	29	14.15
**Age groups (years)**		
>30-39	56	27.32
40–49	69	33.66
50+	80	39.02
**Marital Status**		
Married	173	84.39
Separated	5	2.44
Divorced	6	2.93
Co-habitation	1	0.49
Not married	20	9.76
**Staff rank**		
Full Professor	5	2.44
Associate Professor	31	15.12
Senior Lecturer	76	37.07
Lecturer	75	36.59
Assistant Lecturer	18	8.78
**College**		
Health Sciences	38	18.54
Art and Built Environment	34	16.59
Engineering	34	16.59
College of Science	33	16.1
Agriculture and Natural Resources	33	16.1
Humanities and Social Sciences	33	16.1

### Family history of hypertension and diabetes

A family history of hypertension and diabetes was assessed. We found that 47% and 32% of respondents were aware that family members (either nuclear or extended) had elevated blood pressure and diabetes, respectively. Furthermore, about 64% and 21% of participants mentioned they had been informed they had hypertension and diabetes, respectively.

### Dietary behavior

The majority of participants (62%) take breakfast. About 49.76% of Lecturers consistently take lunch on campus. However, 42.93% are most likely to skip lunch. Carbohydrate makes up the majority of the food content in 173 (84.4%) of the participants with only 11(5.4%) taking more fruits and vegetables in their diets as illustrated in Table 2.

**Table 2:** Dietary behavior among lecturers.

Factors	Freq (n = 205)	Percentage (%)
**Do you have breakfast before the day’s activities on campus?**		
Yes	127	61.95
No	16	7.8
Sometimes	62	30.24
**Do you take lunch on campus during working hours?**		
Yes	102	49.76
No	25	12.2
Sometimes	78	38.05
**Which meals are you most likely to skip?**		
Breakfast	85	41.46
Lunch	88	42.93
Super	32	15.61
**What food content is mostly taken**		
Protein	21	10.24
Carbohydrate	173	84.39
Fruits and Vegetables	11	5.37

### Risk factors for the occurrence of hypertension and diabetes

The study found that 30% of lectures do not undertake any form of exercise, 61.95% undertake mild exercises and 33% do exercises once a week. Moreover, 61% of the lecturers do not consume alcohol and 54% do not often take in soft drinks. Concerning dietary intake, the study found that 34.6% admitted they eat late due to their job and 50% asserted that they sometimes eat late. Moreover, 98% of lecturers do not smoke as shown in Table 3.

**Table 3:** Risk factors for the occurrence of hypertension and diabetes.

Factors	Freq (n = 205)	Percentage (%)
**Exercise**		
**Do you go to the gym or exercise in any other way?**		
Do not undertake any exercise	62	30.24
Undertake mild exercise (1.6–3.0 METS)	127	61.95
Undertake vigorous exercise (6.0 + METS)	16	7.8
**How often do you exercise**		
None	52	25.49
Once a week	68	33.33
Twice a week	42	20.59
More than twice a week	42	20.59
**Alcohol/ Non-Alcoholic Beverage Consumption**		
**How often do you take an alcoholic beverage?**		
None	125	60.98
Not often (once a week)	69	33.66
Often (twice a week)	9	4.39
Very often (3 or more a week)	2	0.98
**Dietary Practices**		
**How often do you take soft drinks?**		
None	56	27.32
Not often (once a week)	110	53.66
Often (twice a week)	30	14.63
Very often (3 or more a week)	9	4.39
**Do you often eat late due to your work?**		
No (before 6 pm)	31	15.12
Yes (after 6 pm)	71	34.63
Sometimes	103	50.24
**Smoking**		
**Do you smoke?**		
Yes	4	1.97
No	199	98.03
**If yes, what do you smoke**		
Cigarette	4	100

### Distribution of lifestyle factors for hypertension using blood pressure of lecturers as the outcome

The study assessed the lifestyle factors associated with the blood pressure outcome of Lecturers. The study found that, among lecturers who do not consume alcohol, 44% have normal BP while 62.57% have high BP. However, among the moderate alcohol consumers, 50% have normal BP and 32.09% have high BP. Among moderate soft drink consumers, 53.5% were found to have high blood pressure. In addition, 35% and 50% of lecturers who regularly or occasionally eat late due to work have high blood pressure as shown in Table 4. All lifestyle factors show no statistical association with BP when the Chi-square test of independence was performed.

**Table 4:** Distribution of accentuating hypertension by blood pressure among participants.

Factors	BP		
	Normal	Hypertension	P-value
**How often do you take an alcoholic beverage?**			0.227
None	8 (44.44)	117 (62.57)	
Not often (moderate)	9 (50.0)	60 (32.09)	
Often to very often	1 (5.56)	10 (5.35)	
**How often do you take soft drinks?**			0.847
None	4 (22.22)	52 (27.81)	
Not often (moderate)	10 (55.56)	100 (53.48)	
Very often	4 (22.22)	35 (18.72)	
**Do you often eat late due to your work?**			0.629
No	4 (22.22)	27 (14.44)	
Yes	5 (27.78)	66 (35.29)	
Sometimes	9 (50.0)	94 (50.27)	
**Do you smoke?**			1.000
Yes	0	4 (2.15)	
No	17 (100)	182 (97.85)	

Statistical significance based on Chi-square or Fishes exact test where appropriate and set at P ≤ 0.05.

### Anthropometric and biochemical characteristics of lecturers

The study assessed the anthropometric and biochemical parameters of Lecturers. About 54.6% of lecturers have high BP (systolic >130 mmHg, diastolic >90 mmHg). Assessment of BMI parameters, weight, and height indicated that 15.6% of lecturers had a healthy weight, while 50.7% and 33.7% were overweight and obese, respectively. With regards to the blood glucose status of Lecturers, it was revealed that 97.07% of lecturers had normal fasting blood glucose (< 5.6 mmol/L) and 85.85% of lecturers had normal random blood sugar (<11.1mmol/dL) levels. The prevalence of hypertension and diabetes was 54.6% and 17.07% respectively. Also, more than half of the study population was recorded as overweight, as shown in Table 5.

**Table 5:** Distribution of anthropometric and biochemical characteristics of participants.

Characteristics	Freq (n = 205)	(%)
**BP (Systolic/Diastolic)**		
Normal (≤120 mmHg or less, ≤ 80 mmHg)	18	8.78
Elevated BP (>120–129 mmHg, > 80 mmHg)	75	36.59
Hypertension (>130 mmHg, > 90 mmHg)	112	54.63
**BMI**		
Underweight (< 18.5 Kg/m^2^)	**–**	–
Healthy weight (18.5 < 25 Kg/m^2^)	32	15.61
Overweight (25.0 < 30 Kg/m^2^)	104	50.73
Obese (>30 Kg/m^2^)	69	33.66
**Fasting Blood Sugar (FBS)**		
Normal (< 5.6 mmol/L)	199	97.07
Prediabetes (5.6–6.7 mmol/L)	5	1.95
Diabetes (≥ 7.0 mmol/L)	1	0.98
**Random Blood Sugar (RBS)**		
Normal (<11.1mmol/dL)	176	85.85
Diabetes (>11.1mmol/dL)	29	14.14

### Sociodemographic factors and blood pressure of lecturers

The study also investigated the association between socio-demographic factors and the blood pressure of Lecturers. It was discovered that 90.91% of male lecturers had elevated BP whereas 93.10% of female lecturers also had elevated BP. Furthermore, there was no significant difference between a lecturers’ age (p = 0.044) and blood pressure. Additionally, of the 174 Lecturers who are not married, separated, or divorced, 90.23% of them had hypertension. Hypertension was identified to be associated (p = 0.036) with lectures across all colleges; the majority (18.54%) of lecturers in the health sciences had very high BP, followed by those in engineering (16.10%), with a minority in Agriculture and Natural Resources (12.68%) shown in Table 6.

**Table 6:** Distribution of sociodemographic factors by blood pressure among participants.

Factors	BP		
	Normal	Hypertension	P-value
**Gender**			0.699
Male	16 (9.09)	160 (90.91)	
Female	2 (6.90)	27 (93.10)	
**Age groups (years)**			**0.044**
< 40	1 (1.79)	55 (98.21)	
40 −49	10 (14.49)	59 (85.51)	
50+	7 (8.75)	73 (91.25)	
**Marital Status**			0.236
Not married/Separated/ Divorced	17 (9.77)	157 (90.23)	
Married/ Co-habitation	1 (3.23)	30 (96.77)	
**Staff rank**			0.491
Full Professor	0	5 (100)	
Associate Professor	2 (6.45)	29 (93.55)	
Senior Lecturer	7 (9.21)	69 (90.79)	
Lecturer	9 (12)	66 (88)	
Assistant Lecturer	0	18 (100)	
**College**			**0.036**
Art and Built Environment	3 (1.46)	31 (15.12)	
Engineering	1 (4.49)	33 (16.10)	
College of Health Sciences	0 (0.0)	38 (18.54)	
Agriculture and Natural Resources	7 (3.41)	26 (12.68)	
Humanities and Social Sciences	4 (1.95)	29 (14.15)	
College of Science	3 (1.46)	30 (14.63)	

Statistical significance based on Chi-square or Fishes exact test where appropriate and set at P ≤ 0.05.

### Dietary behaviour and blood pressure among lecturers

Although none of the factors evaluated showed a statistical association with high BP, the findings showed that 90.55% of lecturers who ate breakfast had hypertension, while 91.18% who did not eat lunch during working hours were also hypertensive. About 90.91% of lecturers identified to be hypertensive reported they were most likely to skip lunch. Most lecturers (91.91%) consumed a lot of carbohydrates in their diets (Table 7).

**Table 7:** Distribution of dietary factors by blood pressure among lecturers.

Factors	BP		
	Normal	Hypertension	P-value
**Do you have breakfast before the day’s activities on campus?**			0.196
Yes	12 (9.45)	115 (90.55)	
No	3 (18.75)	13 (81.25)	
Sometimes	3 (4.84)	59 (95.16)	
**Do you take lunch on campus during working hours?**			0.629
Yes	9 (8.82)	93 (91.18)	
No	1 (4)	24 (96)	
Sometimes	8 (10.26)	70 (89.74)	
**Which meals are you most likely to skip?**			0.645
Breakfast	6 (7.06)	79 (92.94)	
Lunch	8 (9.09)	80 (90.91)	
Super	4 (12.50)	28 (87.50)	
**Food Content is mostly taken**			0.558
Protein	3 (14.29)	18 (85.71)	
Carbohydrate	14 (8.09)	159 (91.91)	
Fruits and Vegetables	1 (9.09)	10 (90.91)	

Statistical significance based on Chi-square or Fishes exact test where appropriate and set at P ≤ 0.05.

### Physical activities and blood pressure among lecturers

The study found that, among those who undertake mild exercises, 62% were found to be hypertensive. Also, the study found that, 26.7% of the lecturers who had not checked their blood pressure before had high blood pressure as shown in Table 8 below.

**Table 8:** Physical activities among lecturers distributed by blood pressure.

Factors	BP		
	Normal	Hypertension	P-value
**Do you go to the gym or exercise in any other way?**			0.851
Do not undertake any exercise	5 (27.78)	57 (30.48)	
undertake mild exercise (1.6–3.0 METS)	11 (61.11)	116 (62.03)	
undertake vigorous exercise (6.0 + METS)	2 (11.11)	14 (7.49)	
**How often do you exercise**			0.332
None	2 (11.76)	50 (26.74)	
Once a week	6 (35.29)	62 (33.16)	
Twice a week	6 (35.29)	36 (19.25)	
More than twice a week	3 (17.65)	39 (20.86)	

### Association between sociodemographic, dietary behaviour and BP among lecturers—Logistic Regression Model

In the bivariate logistic regression model, it was discovered that the age group of 40–49 (p = 0.036) and the affiliation with colleges for lecturers (p = 0.016) are linked with high BP. However, in the multivariate model, only the category of lecturers who were affiliated with colleges in COHS/COS/COE were associated with high BP. In addition, it can be seen from the results that the odds of female lecturers developing high BP is 48% higher compared to male lecturers [AOR: 1.48; 95% CI (0.27–7.96)]. Lecturers from the Colleges of Health, Science and Engineering were 4 times [COR: 4.11; 95% CI (1.30–12.95)] more likely to develop high BP when compared to those in the Colleges of Art and Built Environment, Agriculture and Natural Resources and Humanities and Social Sciences in the bivariate model.

However, in the multivariate model, it was clear that the odd ratio increased to around 5 times higher [AOR: 4.86; 95% CI (1.31–18.06)] among lecturers in the colleges of health, science and engineering than it did among their counterparts from other colleges. The study also revealed that lecturers who do not eat lunch during working hours were more than two times [AOR: 2.6 (0.28–23.72)] more likely to have high BPs compared to those who eat lunch.

Furthermore, it can be noted that lecturers with larger intakes of carbohydrates had odds that are 1.88 times [AOR: 1.88; 95% CI (0.18–19.99)] than those with higher intakes of proteins, Table 9.

**Table 9:** Association between socio-demographic, dietary behaviour, and BP among lecturers—Logistic Regression Model.

	Bivariate		Multivariate	
Factors	COR (95% CI)	P-value	AOR (95% CI)	P-value
**Gender**				
Male	*Ref*		*Ref*	
Female	1.35 (0.29-6.21)	0.700	1.48 (0.27-7.96)	0.650
**Age groups (years)**				
< 40	*Ref*		*Ref*	
40–49	0.11 (0.01-0.87)	**0.036**	0.11 (0.01-1.17)	0.067
50+	0.19 (0.02-1.59)	0.125	0.17 (0.02-1.92)	0.152
**Marital Status**				
Not married/Separated/ Divorced	0.31 (0.04-2.40)	0.261	0.64 (0.07-5.95)	0.693
Married/ Co-habitation	*Ref*		*Ref*	
**Staff rank**				
Full/ Associate Professor	0.56 (0.12-2.56)	0.457	0.48 (0.08-2.81)	0.415
Non-professor	*Ref*		Ref	
**College**				
COHS/COS/COE	4.11 (1.30-12.95)	**0.016**	4.86 (1.31- 18.06)	**0.018**
CABE/CANR/COHSS	*ref*		*ref*	
**Do you have breakfast before the day’s activities on campus?**				
Yes/ Sometimes	*Ref*		*Ref*	
No	0.37 (0.10-1.46)	0.156	0.27 (0.05-1.55)	0.141
**Do you take lunch on campus during working hours?**				
Yes/ Sometimes	*Ref*		*Ref*	
No	2.50 (0.32-19.67)	0.383	2.6 (0.28-23.72)	0.397
**Which meals are you most likely to skip?**				
Breakfast	1.88 (0.49-7.16)	0.354	1.03 (0.19-5.47)	0.969
Lunch	1.43 (0.40-5.11)	0.584	0.80 (0.19-3.34)	0.764
Super	*Ref*		*Ref*	
**Food content is mostly taken**				
Protein	*Ref*		*Ref*	
Fruits and Vegetables	0.60 (0.05-6.56)	0.675	0.39 (0.03-5.57)	0.489
Carbohydrate	1.14 (0.14-9.53)	0.907	1.88 (0.18-19.99)	0.599

OR: Odds Ratio; CI: Confidence Interval; P-value set at 0.05 significance level.

AOR, estimates adjusted for all variables listed in the table. AOR column represents the adjusted estimates from the multivariate model and all included as covariates in the model.

## Discussion

This study sought to evaluate the cardiovascular risk factors of university lecturers in Ghana. We found that close to one-third of respondents had family members who had suffered from hypertension or diabetes. In instances where evidence shows there is the existence of high blood pressure or diabetes among family members individuals may have twice the risk of being diagnosed with high blood pressure or diabetes or both along the life course trajectory, and this conforms to the study results of Nelson & Menna [38]. A family history of hypertension or diabetes significantly increases an individual’s risk of developing these conditions, and a third of respondents having such a history suggests a heightened prevalence of these diseases within their community. This family link indicates a potential genetic predisposition, and also highlights the importance of lifestyle factors and social support in managing and preventing these diseases

Aside from biological disorders, modifiable lifestyle factors such as dietary behavior, excessive alcohol intake, physical inactivity, and excessive tobacco consumption are significantly associated with the occurrence of hypertension and diabetes. Breakfast uptake is essential for the body’s function. Our study reported that a little over half of lecturers take breakfast, while one-third do not consistently take breakfast. This has a positive impact on the health of university lecturers. This is supported by a study conducted at the University of Ohio, which mentioned that people who skip breakfast may miss some major nutrients [39,40]. We realized that among the lecturers interviewed, half of the respondents took lunch during working hours on campus, while a little over one-third were not consistent with lunch uptake. A lack of consistent lunch uptake among lecturers, particularly over one-third, can lead to decreased energy levels, impaired cognitive function, and reduced overall productivity. This can negatively impact teaching quality, student engagement, and potentially affect the lecturers’ own well-being. However, our study finding on lunch uptake is contrary to the study outcome of academics at the University of Kent in Britain, where research on daily records of their activities on the 15th day of each month over a year showed that a little under half of them skipped lunch for various reasons [41].

Our study noted that the majority of the food contents taken by the lecturers are carbohydrates, as against only 5.37% of fruits and vegetables. A diet profoundly skewed towards carbohydrates with minimal fruit and vegetable intake can have several negative health implications. While carbohydrates are a necessary source of energy, an imbalance can lead to weight gain, increased risk of metabolic syndrome, and potentially type 2 diabetes. Equally, a lack of fruit and vegetable intake among lecturers may reduce the intake of essential fiber, minerals, vitamins, and other phytochemicals, which are critical for overall health and disease prevention among lecturers. A search through the literature could not pin down any study implicating university lecturers as high or low fruit and vegetable consumers. However, juxtaposing population-based studies, several reports have consistently indicated a low intake of fruits and vegetables below the recommended consumption of 5 or more servings of fruits or vegetables per day in a typical week [33–44].

Again, our study found that one-third of lecturers do not undertake any form of exercise, while a little over half of the lecturers undertake some mild exercises. This is consistent with the study outcomes of Mohaghegh et. al. [19] where a little over two-thirds of the participants had low physical activity levels and only one-fifth had medium physical activity levels. A significant portion of lecturers not exercising poses several health and performance risks, while the “mild exercise” group might not be reaping the full benefits of physical activity. The implications range from increased risk of chronic diseases to potential negative impacts on cognitive function and overall well-being. Lack of physical activity is a major risk factor for obesity, type 2 diabetes, cardiovascular diseases, and some types of cancer. Exercise is crucial for maintaining a healthy heart and blood vessels. The majority of lecturers not exercising can lead to a decline in cardiovascular fitness. fatigue and decreased overall energy levels. It is therefore not surprising to note that over half of our respondents mentioned they had been informed they have hypertension, and 21% mentioned they have been told they have diabetes. This outcome aligns well with the study by Marcio et. al. [21] who reported that a low number of lecturers performed regular physical activities so and a little under half of lecturers were at high risk of cardiovascular diseases.

The alcohol consumption patterns among lecturers at Kwame Nkrumah University of Science and Technology (KNUST) are generally positive, with a significant proportion not consuming alcohol at all, and another substantial portion being infrequent consumers. This suggests a relatively healthy overall trend, but it’s crucial to understand the implications of even moderate alcohol use on academic and professional life of lecturers. Alcohol consumption, even at moderate levels, can have negative effects on academic performance, including absenteeism, difficulty completing tasks, and reduced productivity. It can also affect lecturers’ health, potentially leading to disorganization, ill health, and a less productive work environment. Barring all other confounders, it is believed that alcohol-related diseases may be minimal among lecturers in KNUST. However, our finding is contrary to the study outcomes of Awoliyi et. al. [27] who discovered that 39% of academic respondents were habitual alcohol consumers in a UK University. Similarly, we also recorded contrary results from the study outcome of Klein et al. [20], who found that the majority of physician faculty members consume alcohol. However, our study noted that, of the lecturers who are moderate alcohol consumers and habitual alcohol consumers, one-third of the sample and very few are hypertensive, respectively (see Table 4). The existence of these two conditions is not too good for the holistic health of lecturers in an academic institution.

We further inquired about the frequency of soft drink consumption among lecturers on campus. In our Ghanaian parlance, soft drinks are typically drinks with high sugar content, such as Coca-Cola, Fanta, Malta, Maltina, Pepsi Cola, Sprite, and a host of other soft drinks available in markets in Ghana. Our study, however, noted that over half of the lecturers took soft drinks, though not often. As half of university lecturers are noted of taking soft drinks, it could have several implications, primarily concerning health. Soft drinks are often high in sugar and calories, contributing to weight gain and increasing the risk of obesity. Obesity, in turn, is linked to a higher risk of type 2 diabetes, heart diseases, and other chronic conditions. A search through the literature did not yield any complementary or contrary study reporting on soft drink consumption among university lecturers. However, a critical look at the cumulative numbers of soft drink consumers (those who take soft drinks often and those who do not take soft drinks often) revealed that 72.20% of the lecturers in this bracket were hypertensive (see Table 4). The already fragile health conditions of these lecturers may be exacerbated by certain ingredients in soft drinks, which may be hazardous to health if consumed in large quantities and over sustained period of time [45]. For instance, research outcomes have demonstrated that most soft drinks contain phosphorus. A high level of phosphorus in the blood (“hyperphosphatemia”) can lead to kidney diseases and vice-versa, and subsequently lower calcium levels with associated risk of brittle bone and cardiovascular diseases [45,46].

The discovery that one-third to one-half of lecturers are habitual late eaters has several significant implications. It suggests potential health risks, including increased risk of type 2 diabetes and cardiovascular issues due to disruptions in glucose metabolism. It could also indicate a link between late eating and unhealthy eating habits, potentially leading to weight gain. Furthermore, it highlights a need to address the factors, both personal and environmental, that contribute to this trend among lecturers. A search through the literature did not yield any corresponding studies reporting on the eating time of lecturers. However, community-based studies reporting on the negative impact of eating late were found [47–49]. For instance, Vujović, [48] found that eating four hours later makes a significant modification in hunger levels, burning calories after meals, and fat storage. Also, Reynolds [49] mentioned that late eating may have implications for the occurrence of conditions such as weight gain, diabetes, increased body fat, and impaired weight loss success. Conspicuously, these findings transport physiological and molecular mechanisms fundamental to the association between late eating and increased obesity risk with implications for diabetes and hypertension. Perhaps this may explain why about one-third of the late eaters and half of the inconsistently late eaters have hypertension *(see* Table 4*, late eating variable).* Equally, our study recorded one-third and a little over half of lecturers as having elevated BP (>120, < 129 mmHg, > 80 mmHg or less) and hypertension (>130 mmHg, > 90 mmHg) respectively *(see* Table 5*).* Again, our data also reveals that half of the lecturers are overweight (>25.0 > 30 kg/m^2^) and one-third are obese (>30 kg/m^2^) which are clear indications of the negative effects of late eating, though other factors may be contributors *(see* Table 5*).* This result is consistent with a study conducted in a large urban university where close to half of the respondents were classified as obese [50]. The results are also consistent with a cross-sectional study from the Amazon Basin where poor cardiovascular health was found in almost all the University Professors [51].

Moreover, our study also assessed the smoking status of lecturers. We found that the overwhelming majority of the lecturers do not smoke in any form. This is in line with the study outcome of Owusu-Dabo et. al., [25] who reported that smoking is prevalent among people of low socio-economic status. A similar study reported by Klein et al. [20] found smoking to be rare among the physician faculty. The fact that the overwhelming majority of lecturers do not smoke cigarettes or any form can have several positive implications for lecturers’ health, students and the institution. It can reduce secondhand smoke exposure, potentially improve the overall academic environment, and serve as a positive role model for students. Smoking can negatively affect academic performance, and non-smoking role models can influence students’ decisions.

However, it can be seen from the results (*see* Table 9) that, the odds of female lecturers developing high BP is 48% higher compared to male lecturers [AOR: 1.48; 95% CI (0.27–7.96)]. A 48% higher risk of high blood pressure (hypertension) in female lecturers compared to male lecturers suggests a significant gender disparity in cardiovascular health within this specific profession. This difference highlights the need for targeted interventions and awareness campaigns to address potential risk factors and promote healthier lifestyles among female academics. This is similar to the study outcome of Alinaitwe et al [12], which reported of higher prevalence of hypertension among female lecturers in an urban university. More studies are needed to investigate the specific mechanisms underlying gender differences in hypertension among lecturers and to develop effective prevention and management strategies. However, our result is contrary to population-based studies, which reported higher odds of males developing hypertension than females [52,53].

The study found a significant difference in the age category of lecturers (p = 0.044) across the study participants. Studies linking university lecturer age categories with hypertension reveal a higher prevalence of hypertension in older age groups [12,50,54–56]. This suggests that age is a significant risk factor for hypertension, with older lecturers experiencing higher blood pressure compared to their younger counterparts. However, our study could not establish any significant link between a lecturers age and occurrence of diabetes and this can be explained by other confounders outside the scope of this study.

## Conclusion

The study titled “Assessment of Hypertension and Diabetes among Lecturers in a Ghanaian University - Setting Lifestyle Practices as Precursors” explores the relationship between lifestyle practices and the prevalence of hypertension and diabetes among university lecturers in Ghana. The study employs a cross-sectional design involving 205 lecturers and analyzes data using descriptive statistics and regression methods. Key findings reveal that a significant proportion of lecturers are either overweight or obese, with a concerning number not engaging in regular exercise. Age is a statistically significant factor associated with increased blood pressure, particularly among those aged 50 and above. The study concludes that modifiable lifestyle practices are key precursors for the occurence of hypertension and diabetes among this population.

## Recommendations

The findings of this study underscore the need for targeted, multi-component interventions at the institutional and individual level to address modifiable lifestyle practices among lecturers. Recommendations are focused on promoting physical activity, healthy nutrition, and regular health monitoring.

### Institutional level recommendations

1. **Comprehensive workplace wellness programs:** The university administration should develop and implement multi-component programs that include both nutrition and physical activity strategies. These can be more effective with support from university authorities and multidisciplinary health professionals.
2. **Provide access to fitness facilities and programs:** Offer subsidized or free on-campus gym memberships, strengthen group exercise classes such as “keep fit” clubs, and create walking/ jogging paths to ensure safety and thereby encouraging regular physical activity
3. **Integrate physical activity into the workday:** Encourage short physical activity breaks during long meetings or sedentary work periods. Policies could be developed to allow for flexible schedules that accommodate exercise time.
4. **Promote healthy eating environments:** Subsidize healthy food options in university cafeterias focusing on fruits, vegetables, and whole grains, while limiting high-fat and high-sugar items.
5. **Counselling Services:** Provide nutrition counseling to improve dietary practices.
6. **Offer regular health screenings and counseling:** Reduce the current annual, confidential health screenings for blood pressure, BMI, cholesterol, and blood glucose levels to every six (6) months. This helps in early detection and provides opportunities for health professionals to offer personalized lifestyle modification advice.
7. **Targeted interventions for high-risk groups:** Develop specific interventions for lecturers aged 50 and above, as age is a significant factor associated with increased blood pressure and diabetes. These programs can include close monitoring and personalized guidance.
8. **Raise awareness through education:** Conduct workshops and awareness campaigns electronic text-massage pop-ups, emails and university local radio and TV stations to educate lecturers on the risks associated with obesity, physical inactivity, hypertension, and diabetes. The awareness creation should emphasize that these conditions are often linked to modifiable lifestyle factors.

### Individual-level recommendations

1. **University lecturers adhere to physical activity guidelines:** Lecturers should aim for at least 150 minutes of moderate-intensity aerobic activity or 75 minutes of vigorous activity per week, in addition to muscle-strengthening exercises.
2. **University lecturers adopt healthy dietary practices:** Focus on a balanced diet, potentially following approaches like the DASH (Dietary Approaches to Stop Hypertension) diet, which emphasizes reduced sodium and increased intake of potassium, magnesium, fruits, and vegetables.
3. **Manage weight:** Individuals who are overweight or obese should be encouraged to pursue modest weight loss goals, as even small reductions can lower blood pressure and diabetes risk.
4. **Seek professional advice:** Consult healthcare professionals for personalized advice on managing blood pressure, developing an exercise and nutritional plans and addressing weight concerns.

The study hopes that, by implementing these multi-faceted recommendations, the universities can foster a healthier environment, reduce the prevalence of modifiable risk factors, and ultimately improve the long-term health outcomes of lecturers.

### Study limitations

This study is limited in scope due to the inability to reach the intended sample size due to budget constraints. Again, there is a limitation in scope relating to external validity due to the single-center design. Also, the 3 times of short period blood pressure measurement on the same day were also enough to make declaration of hypertension status. Equally, the onetime glucose levels used was also a limitation. Despite these limitations, the researchers believe that the outcomes from the study may serve as baseline data for future research and prompt university authorities to employ robust measures to check the hypertension and diabetes status of lecturers.

## Direction of future study

Future studies should involve more than three universities in Ghana so that the various parameters emerging out of the study can be generalized.

## Abbreviations

AOR: Adjusted Odds Ratio
BMI: Body Mass Index
CABE: College of Art & Built Environment
CANR: College of Agric and Natural Resources
CHRPE: Committee on Human Research, Publication and Ethics
COE: College of Engineering
COHS: College of Health Sciences
COHSS: College of Humanities & Social Sciences
COS: College of Science
KNUST: Kwame Nkrumah University of Science and Technology
NCDs: Non-communicable Diseases
OR: Odds Ratio.

## References

[1] Antonovsky A. Health, stress, and coping. San Francisco: Jossey-Bass Publishers; 1979.

[2] Mittelmark M, Bauer G. The Meanings of Salutogenesis. In: The Handbook of Salutogenesis. 2017. p. 7–13. 28590655

[3] Fries CJ. Healing Health Care: From Sick Care Towards Salutogenic Healing Systems. Soc Theory Health. 2020;18(1):16–32. doi: 10.1057/s41285-019-00103-2 32226316 PMC7099730

[4] Espnes GA, Monknes UK, Haugan G. The Overarching Concept of Salutogenesis in the Context of Health Care. In: Haugan G, Eriksson M, editors. Health Promotion in Health Care-Vital Theories and Research. Cham: Springer; 2021. 10.1007/978-3-030-63135-2_2 36315731

[5] Tagoe HA, Dake FA. Healthy lifestyle behaviour among Ghanaian adults in the phase of a health policy change. Global Health. 2011;7:7. doi: 10.1186/1744-8603-7-7 21473779 PMC3090331

[6] Heidari M, Borujeni MB, Borujeni MG, Shirvani M. Relationship of Lifestyle with Academic Achievement in Nursing Students. J Clin Diagn Res. 2017;11(3):JC01–3. doi: 10.7860/JCDR/2017/24536.9501 28511411 PMC5427337

[7] Mushemeza ED. Opportunities and Challenges of Academic Staff in Higher Education in Africa. Int J Higher Educ. 2016;5(3). doi: 10.5430/ijhe.v5n3p236

[8] Meng Q, Wang G. A research on sources of university faculty occupational stress: a Chinese case study. Psychol Res Behav Manag. 2018;11:597–605. doi: 10.2147/PRBM.S187295 30573995 PMC6292231

[9] Cristiani V, Kumbamu A, Asiedu GB, Johnson SK, Gewirtz O’Brien JR, Ziebart G, et al. Use of Community Based Participatory Research to Design Interventions for Healthy Lifestyle in an Alternative Learning Environment. J Prim Care Community Health. 2021;12:21501327211014749. doi: 10.1177/21501327211014749 33980061 PMC8127794

[10] Akoto S, Tandoh MA, Nsiah K, Asamoah-Boakye O, Annaful VT. Lifestyle habits, macronutrient intake, and obesity prevalence among adolescents in rural-periurban community senior high schools in the Ho municipality of Ghana. Front Nutr. 2022;9:955898. doi: 10.3389/fnut.2022.955898 36110405 PMC9468859

[11] Brenyah JK, Kyei-Dompim J, Koranteng Tannor E, Twum P, Boakye Okyere P, Gyapong-Korsah B, et al. Assessment of non-communicable diseases screening practices among university lecturers in Ghana – a cross sectional single centre study. F1000Res. 2023;12:746. doi: 10.12688/f1000research.134627.2

[12] Alinaitwe B, Amanya C, A Muwanguzi P, Ngabirano TD. Prevalence of Risk Factors for Hypertension Among Faculty at an Urban University in Uganda. Integr Blood Press Control. 2024;17:1–11. doi: 10.2147/IBPC.S440972 38196839 PMC10773241

[13] Omorogiuwa A, Oaikhena GA, Okioya P, Akubueze D, Owobu E, Enahoro I, et al. Diabetes Mellitus: Prevalence Amongst University Staff In Southern Nigeria And Attitude Towards Routine Glycemic/Glucosuric Checkup. Int J Biomed Health. 2009;6.

[14] Farhud DD. Impact of lifestyle on health. Iran J Public Health. 2015;44(11):1442–4. 26744700 PMC4703222

[15] Jarosz E. Lifestyle behaviours or socioeconomic characteristics? Gender differences in covariates of BMI in Hungary. Obes Sci Pract. 2018;4(6):591–9. doi: 10.1002/osp4.316 30574352 PMC6298311

[16] Magalhães C, Ribeiro MF, Esteves MR, Aires L, Lima S, Silva G, et al. Behavioral profile, lifestyle and social skills in Portuguese adolescents. BMC Public Health. 2021;21(1):384. doi: 10.1186/s12889-021-10355-1 33602184 PMC7893771

[17] Kwasi BJ, Koranteng TE, Florence B, Anthony E. Political economy framework and the occurrence of noncommunicable diseases. “Framing dietary practices in Ghana as the receptacle”. Int J Noncommun Dis. 2021;6(3):122–8. doi: 10.4103/jncd.jncd_30_21

[18] Sharma S, Matheson A, Lambrick D, Faulkner J, Lounsbury DW, Vaidya A, et al. Dietary practices, physical activity and social determinants of non-communicable diseases in Nepal: A systemic analysis. PLoS One. 2023;18(2):e0281355. doi: 10.1371/journal.pone.0281355 36745612 PMC9901760

[19] Mohaghegh S. Physical activity among Iranian physicians and faculty members: A cross sectional study. Biosci Biotech Res Comm. 2017;10(4):805–9. doi: 10.21786/bbrc/10.4/29

[20] Klein D, Guenther C, Ross S. Do as I say, not as I do: lifestyles and counseling practices of physician faculty at the University of Alberta. Can Fam Physician. 2016;62(7):e393-9.

[21] Márcio A, Cauterrucio Â, Renan F, Magda G, Mercedes N, Tatiana R, Donizete V. Occupational health and university professors’ lifestyle: Integrative literature review. UERJ. 2021;29:e60812. doi: 10.12957/reuerj.2021.60812

[22] Morris E, Unwin N, Ali E, Brathwaite-Graham L, Samuels TA. Chronic non-communicable disease risk factor survey 2010 among University of the West Indies staff at Cave Hill, Barbados. West Indian Med J. 2011;60(4):452–8. 22097677

[23] Singh A, Owusu-Dabo E, Mdege N, McNeill A, Britton J, Bauld L. A situational analysis of tobacco control in Ghana: progress, opportunities and challenges. J Glob Health Rep. 2020;4:e2020015. doi: 10.29392/001c.12260 33184609 PMC7116349

[24] Dai X, Gakidou E, Lopez AD. Evolution of the global smoking epidemic over the past half century: strengthening the evidence base for policy action. Tob Control. 2022;31(2):129–37. doi: 10.1136/tobaccocontrol-2021-056535 35241576

[25] Owusu-Dabo E, Lewis S, McNeill A, Gilmore A, Britton J. Smoking uptake and prevalence in Ghana. Tob Control. 2009;18(5):365–70. doi: 10.1136/tc.2009.030635 19581276 PMC2745559

[26] Home Office-UK. The Government’s Alcohol Strategy. 2012 [cited on 1st December, 2022]. Available from: http://www.official-documents.gov.uk/document/cm83/8336/8336.pdf

[27] Awoliyi S, Ball D, Parkinson N, Preedy VR. Alcohol misuse among university staff: a cross-sectional study. PLoS One. 2014;9(7):e98134. doi: 10.1371/journal.pone.0098134 25072628 PMC4114484

[28] Kuppersmith N, Kennedy C. Perils of Skipping Meals. University of Louisville Research Foundation Inc. 2005 [cited on 18th November, 2022]. Available from: https://louisville.edu/medicine/departments/familymedicine/files/L081611.pdf

[29] Song WO, Chun OK, Obayashi S, Cho S, Chung CE. Is consumption of breakfast associated with body mass index in US adults? J Am Diet Assoc. 2005;105(9):1373–82. doi: 10.1016/j.jada.2005.06.002 16129078

[30] Mekary RA, Giovannucci E, Willett WC, van Dam RM, Hu FB. Eating patterns and type 2 diabetes risk in men: breakfast omission, eating frequency, and snacking. Am J Clin Nutr. 2012;95(5):1182–9. doi: 10.3945/ajcn.111.028209 22456660 PMC3325839

[31] Koene RJ, Prizment AE, Blaes A, Konety SH. Shared Risk Factors in Cardiovascular Disease and Cancer. Circulation. 2016;133(11):1104–14. doi: 10.1161/CIRCULATIONAHA.115.020406 26976915 PMC4800750

[32] Habib A, Alam MM, Hussain I, Nasir N, Almuthebi M. Erratic Behavioral Attitude Leads to Noncommunicable Diseases: A Cross-Sectional Study. Biomed Res Int. 2020;2020:7257052. doi: 10.1155/2020/7257052 32420366 PMC7199604

[33] Cena H, Calder PC. Defining a Healthy Diet: Evidence for The Role of Contemporary Dietary Patterns in Health and Disease. Nutrients. 2020;12(2):334. doi: 10.3390/nu12020334 32012681 PMC7071223

[34] López-Olivares M, De Teresa Galván C, Nestares T, Fernández-Gómez E, Enrique-Mirón C. Lifestyle Factors Influencing Dietary Patterns of University Professors. Int J Environ Res Public Health. 2021;18(18):9777. doi: 10.3390/ijerph18189777 34574700 PMC8472133

[35] Hulsegge G, Proper KI, Loef B, Paagman H, Anema JR, van Mechelen W. The mediating role of lifestyle in the relationship between shift work, obesity and diabetes. Int Arch Occup Environ Health. 2021;94(6):1287–95. doi: 10.1007/s00420-021-01662-6 33704584 PMC8292292

[36] KNUST. Quality Assurance and Planning Unit, KNUST-Kumasi. 2020.

[37] Yamane Y. Mathematical Formulae for Sample Size Determination. 1967 [cited 19th December 2022]. Available from: https://www.scirp.org/reference/referencespapers?referenceid=2995457

[38] Nelson C, Menna M. Genetic Risk Factors for High Blood Pressure. 2022 [cited 24th November 2022]. Available from: https://www.verywellhealth.com/high-blood-pressure-genes-5270333

[39] Fanelli S, Walls C, Taylor C. Skipping breakfast is associated with nutrient gaps and poorer diet quality among adults in the United States. Proc Nutr Soc. 2021;80(OCE1). doi: 10.1017/s0029665121000495

[40] Caldwell E. Adults who skip morning meal likely to miss out on nutrients Science Daily. 2021 [cited on 28th November, 2022]. Available from: https://www.sciencedaily.com/releases/2021/06/210615132207.htm

[41] Fincher S, Dziallas S, Havergal C. No Time for Lunch? Times Higher Education. 2015 [cited on 2nd December, 2022]. Available from: https://www.insidehighered.com/news/2015/12/17/study-finds-british-academics-skip-lunch

[42] Sabzghabaee AM, Mirmoghtadaee P, Mohammadi M. Fruit and Vegetable Consumption among Community Dwelling Elderly in an Iranian Population. Int J Prev Med. 2010;1(2):98–102. 21566769 PMC3075478

[43] Amo-Adjei J, Kumi-Kyereme A. Fruit and vegetable consumption by ecological zone and socioeconomic status in Ghana. J Biosoc Sci. 2015;47(5):613–31. doi: 10.1017/S002193201400025X 24991697

[44] Kabwama SN, Bahendeka SK, Wesonga R, Mutungi G, Guwatudde D. Low consumption of fruits and vegetables among adults in Uganda: findings from a countrywide cross-sectional survey. Arch Public Health. 2019;77(1). doi: 10.1186/s13690-019-0332-6 PMC6366031 30774951

[45] Kregiel D. Health safety of soft drinks: contents, containers, and microorganisms. Biomed Res Int. 2015;2015:128697. doi: 10.1155/2015/128697 25695045 PMC4324883

[46] Martin KJ, González EA. Prevention and control of phosphate retention/hyperphosphatemia in CKD-MBD: what is normal, when to start, and how to treat?. Clin J Am Soc Nephrol. 2011;6(2):440–6. doi: 10.2215/CJN.05130610 21292848

[47] Kinsey AW, Ormsbee MJ. The health impact of nighttime eating: old and new perspectives. Nutrients. 2015;7(4):2648–62. doi: 10.3390/nu7042648 25859885 PMC4425165

[48] Vujović N. Late-night eating and weight gain. The Harvard Gazette. Harvard University; 2022 [cited on 24th November 2022]. Available online: https://news.harvard.edu/gazette/story/2022/10/study-looks-at-why-late-night-eating-increases-obesity-risk/

[49] Reynolds W. Why late-night eating leads to weight gain, and diabetes. Northwestern University Journal. 2022 [cited on 3rd December 2022]. Available from: https://news.northwestern.edu/stories/2022/10/why-late-night-eating-leads-to-weight-gain-diabetes/

[50] Tamuno-O A, Stanley RO, Amadi-Ikpa H, Ajulor EK, Sarah DB, Tamuno-Opubo M. Awareness of hypertension, risk factors and its complications among university lecturers in Rivers State, Nigeria. J Med Dental Sci Res. 2023;10(3):01–9.

[51] Marjorie RF, Rebecca J, Rubinstein MS. Obesity and Food Choices Among Faculty and Staff at a Large Urban University, J Am College Health. 2010;59(3):205–10. doi: 10.1080/07448481.2010.502203 21186451

[52] Muniz DD, Siqueira KS, Cornell CT, Fernandes-Silva MM, Muniz PT, Silvestre OM. Ideal Cardiovascular Health and Job Strain: A Cross-Sectional Study from the Amazon Basin. Arq Bras Cardiol. 2019;112(3):260–8. doi: 10.5935/abc.20190005 30916204 PMC6424039

[53] Dosoo DK, Nyame S, Enuameh Y, Ayetey H, Danwonno H, Twumasi M, et al. Prevalence of Hypertension in the Middle Belt of Ghana: A Community-Based Screening Study. Int J Hypertens. 2019;2019:1089578. doi: 10.1155/2019/1089578 31687204 PMC6800906

[54] Atibila F, Hoor GT, Donkoh ET, Wahab AI, Kok G. Prevalence of hypertension in Ghanaian society: a systematic review, meta-analysis, and GRADE assessment. Syst Rev. 2021;10(1):220. doi: 10.1186/s13643-021-01770-x 34364395 PMC8349493

[55] Defianna SR, Santosa A, Probandari A, Dewi FST. Gender Differences in Prevalence and Risk Factors for Hypertension among Adult Populations: A Cross-Sectional Study in Indonesia. Int J Environ Res Public Health. 2021;18(12):6259. doi: 10.3390/ijerph18126259 34207848 PMC8296037

[56] Calvo MS, Tucker KL. Is phosphorus intake that exceeds dietary requirements a risk factor in bone health? Ann N Y Acad Sci. 2013;1301:29–35. doi: 10.1111/nyas.12300 24472074

